# Point-of-Care Ultrasound to Diagnose Molar Pregnancy: A Case Report

**DOI:** 10.21980/J82W7T

**Published:** 2022-04-15

**Authors:** Katherine Wietecha, Caitlin A Williams, Valori Slane

**Affiliations:** * Kendall Regional Medical Center, Department of Emergency Medicine, Miami, FL; ^ Nova Southeastern University, Dr. Kiran C Patel College of Allopathic Medicine, Fort Lauderdale, FL

## Abstract

Molar pregnancies are rare complications that can have potentially devastating effects, including neoplastic disease. Given the potential for malignant conversion, proper diagnosis of molar pregnancy is crucial. This case demonstrates the utility of point-of-care ultrasound (POCUS) for molar pregnancy in the emergency department (ED). The patient was a 43-year-old G8P1, 8-week-pregnant female who presented to the emergency department for evaluation of abdominal pain. Her physical exam was notable for abdominal tenderness and a normal pelvic exam. Evaluation included basic labs with a quantitative serum beta human chorionic gonadotropin (βhCG), urinalysis, and vaginal wet mount. Her results were unremarkable with the exception of elevated βhCG to 83,000 mIU/mL. A transabdominal POCUS was performed which showed a heterogeneous mass with several anechoic areas, concerning for a molar pregnancy. Patient was seen by obstetrics and gynecology (OB-GYN) and a transvaginal ultrasound showed similar findings. Dilation and evacuation were performed approximately 5 hours after initial diagnosis on POCUS. Use of POCUS was crucial for expedient diagnosis and appropriate treatment in this patient, highlighting the utility of POCUS for pregnant patients in the ED.

**Topics:**

Molar pregnancy, gestational trophoblastic disease, hydatidiform mole, point of care ultrasound.

**Figure:**
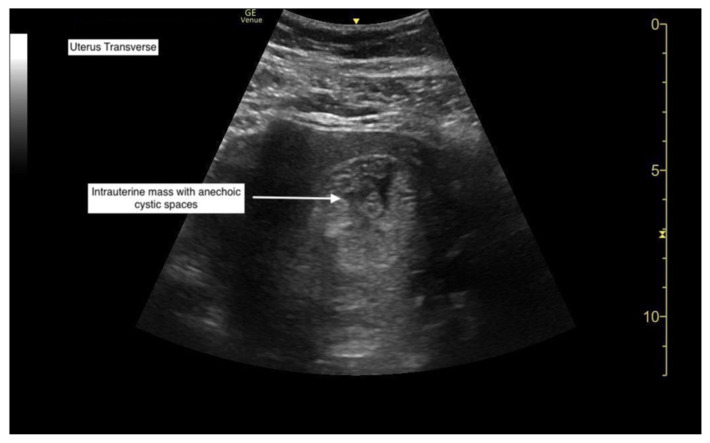
Video Link: https://youtu.be/fKvetUnQ15s

## Brief introduction

Molar pregnancies typically present in the first trimester as the result of abnormal proliferation of placental trophoblastic tissue. In the United States, incidence estimates suggest that molar pregnancies occur at a rate of approximately 1 per 1000 pregnancies.^1^ Women of advanced maternal age have been shown to be at increased risk compared to women considered to be of average maternal age.^2^,^3^ However, it has been shown that Hispanic women tend to be diagnosed with molar pregnancies at earlier ages than other ethnicities.^4^ Furthermore, women of advanced maternal age are at increased risk for malignant changes, eventually requiring chemotherapy.^3^

Diagnosis of molar pregnancy begins with measuring quantitative levels of serum beta human chorionic gonadotropin (βhCG), which are elevated, approximately 100,000 mIU/mL, in molar pregnancy.^1^ Complete molar pregnancy can be visualized on ultrasound as a heterogeneous mass with a snowstorm appearance, with the absence of fetal development.^5^ Historically, it has been difficult to diagnose molar pregnancy on ultrasound; recently, there have been improvements in sensitivity, making point-of-care ultrasound (POCUS) a key instrument in diagnosis.^6^

## Presenting concerns and clinical findings

A 43-year-old G8P1, 8-weekpregnant female presented to the ED with lower abdominal pain for fifteen days. She had no other associated symptoms. She had recently presented to an outside OB-GYN clinic for an elective abortion and was told her ultrasound showed an abnormal finding. The patient was told the abortion could not be completed due to the abnormal finding. The patient was not made aware of the specifics of the abnormality. Her last menstrual period was eight weeks prior to her presentation to the ED. Her initial triage vitals were unremarkable. On physical exam, she had mild abdominal tenderness to palpation and an unremarkable pelvic exam.

## Significant findings

Her βhCG was found to be 83,000 mIU/mL. A transabdominal point-of-care ultrasound (POCUS) was initiated to determine whether an abnormality to the pregnancy could be identified. Curvilinear probe was used. Our transabdominal POCUS, in the transverse plane, showed a heterogenous mass with multiple anechoic areas in the uterus. The white arrow on the ultrasound identifies these findings. The classic “snowstorm” appearance was concerning for molar pregnancy.

## Patient course

Upon arrival at the ED, the patient’s vitals were stable and her physical exam remarkable for abdominal tenderness. Transabdominal POCUS showed an intrauterine mass concerning for a molar pregnancy. Transvaginal ultrasound was ordered to evaluate for ectopic pregnancy. Labs, urine analysis, and wet mount ruled out other causes of the patient’s abdominal pain.

Consultation to OB-GYN and gynecologic oncology was expedited due to our POCUS. The formal transvaginal ultrasound confirmed a molar pregnancy. The patient was admitted to the hospital and taken to the operating room for a dilation and evacuation within five hours of admission. The patient’s dilation and evacuation were completed, and the patient was discharged home the same day as her presentation to the ED. The pathology report from the dilation and evacuation was consistent with a complete hydatidiform mole. No fetal tissue was identified from the specimen. The patient had a follow-up with her gynecologic oncologist seven days after her operation.

## Discussion

Care in the emergency department can sometimes be the first encounter pregnant patients have, particularly in early pregnancy.^7^ As such, it is necessary that emergency medicine physicians utilize point-of-care ultrasound to ensure timely management.

Patients with molar pregnancy typically present with vaginal bleeding or abdominal pain,^8^,^9^ as was the case with our patient. Criteria for diagnosis includes elevated quantitative levels of serum βhCG beyond what would be expected for gestational age.^1^ Typically, complete molar pregnancies are diagnosed with βhCG above 100,000 and incomplete or partial molar pregnancies having levels below 100,000.^1^ Ultrasound and eventual pathological evaluation of the pregnancy provide information to make a definitive diagnosis.^1^

Although molar pregnancies are typically identified via transvaginal ultrasound, this case shows that point-of-care transabdominal ultrasounds can be used in the emergency department to improve diagnosis of molar pregnancy. Ultrasound findings of complete molar pregnancies include an abnormal gestational sac containing a central heterogeneous mass with anechoic spaces that lacks fetal parts.^1^,^8^ The classic snowstorm appearance of the placenta in complete moles is typically not visualized in the first trimester^1^, as it was in this patient. Theca lutein cysts can also be visualized in complete molar pregnancies of later gestational age.^1^

Follow-up for molar pregnancy is crucial given the increased risk of progression to malignant gestational trophoblastic neoplasia.^1^ As such, expedient treatment after diagnosis is necessary, with options including chemotherapy, dilation and curettage, or hysterectomy, depending on the extent of the molar pregnancy and malignant characteristics.^10^ These serious complications further support the use of point-of-care ultrasound to hasten diagnosis and treatment.

## Supplementary Information






