# Supplementary figures and images for: Point-of-Care Ultrasound to Diagnose Molar Pregnancy: A Case Report
Source: J Educ Teach Emerg Med. 2022 Apr 15;7(2):V1–3. doi: 10.21980/J82W7T (PMC10332747; doi:10.21980/J82W7T)

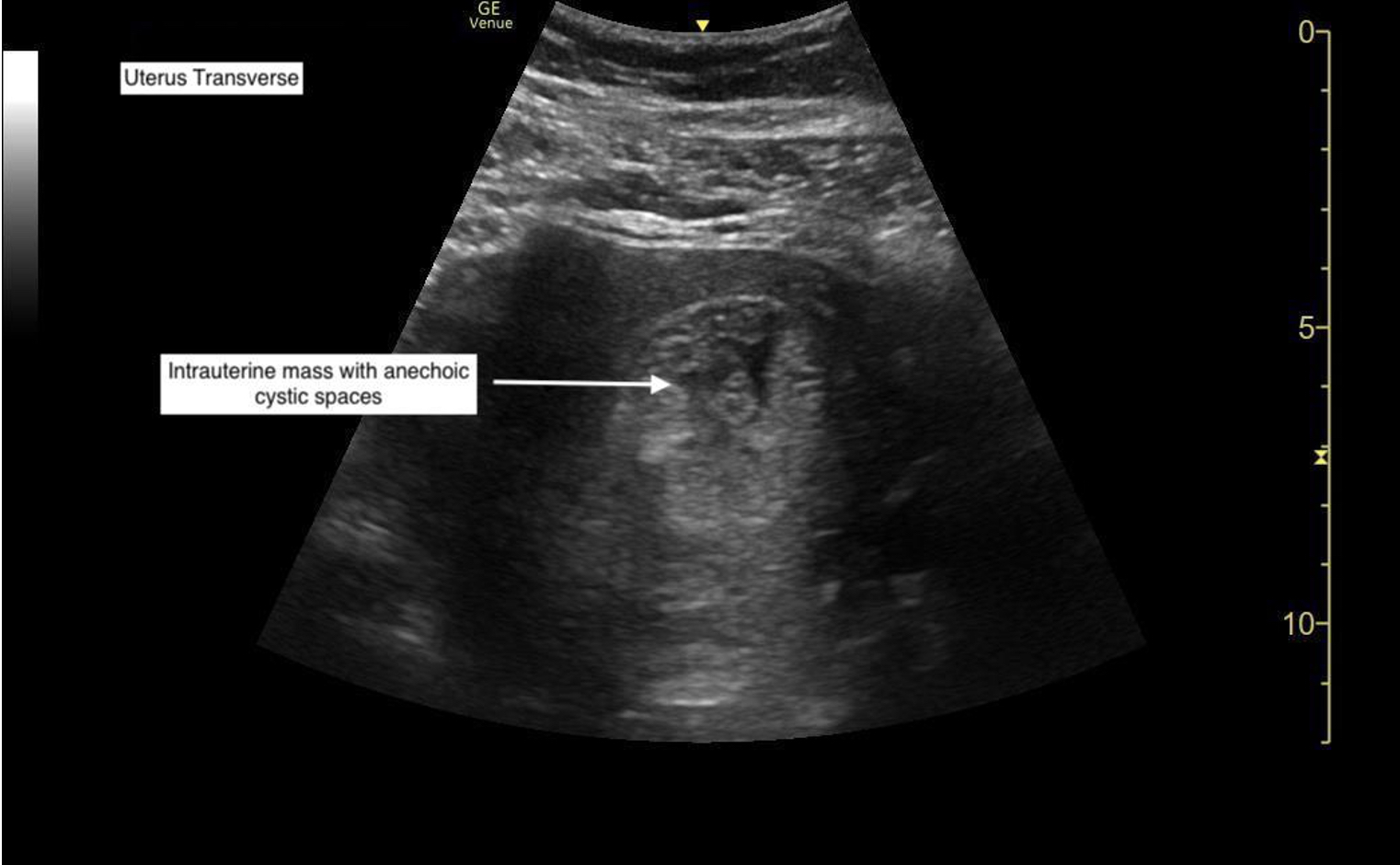

Supplement: Supplementary file 1 [file JETem-7-2-V1-supp1.jpg]

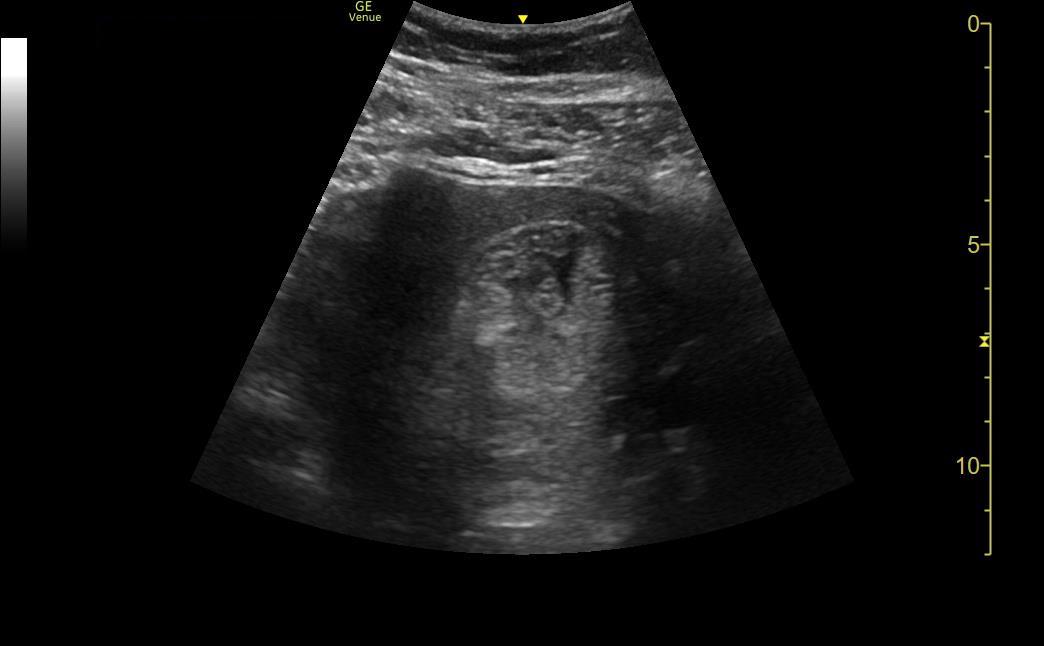

Supplement: Supplementary file 2 [file JETem-7-2-V1-supp2.jpg]
